# Trends in Mortality after Intensive Care of Patients with Aneurysmal Subarachnoid Hemorrhage in Finland in 2003–2019: A Finnish Intensive Care Consortium study

**DOI:** 10.1007/s12028-021-01420-z

**Published:** 2021-12-29

**Authors:** Jyri J. Virta, Markus Skrifvars, Matti Reinikainen, Stepani Bendel, Ruut Laitio, Sanna Hoppu, Tero Ala-Kokko, Jari Siironen, Rahul Raj

**Affiliations:** 1 Department of Neurosurgery, Helsinki University Hospital, University of Helsinki, Helsinki, Finland; 2 Division of Anesthesiology, Department of Anesthesiology, Intensive Care and Pain Medicine, Helsinki University Hospital, University of Helsinki, Helsinki, Finland; 3 Department of Emergency Care and Services, Helsinki University Hospital, University of Helsinki, Helsinki, Finland; 4 Department of Anesthesiology and Intensive Care, Kuopio University Hospital, University of Eastern Finland, Kuopio, Finland; 5 Department of Perioperative Services, Intensive Care and Pain Management, Turku University Hospital, University of Turku, Turku, Finland; 6 Department of Intensive Care and Emergency Medicine Services, Department of Emergency, Anesthesia and Pain Medicine, Tampere University Hospital, University of Tampere, Tampere, Finland; 7 Research Group of Surgery, Anesthesiology and Intensive Care, Division of Intensive Care, Oulu University Hospital, University of Oulu, Medical Research Center, Oulu, Finland

**Keywords:** Subarachnoid hemorrhage, Critical care outcomes

## Abstract

**Background:**

Previous studies suggest that case mortality of aneurysmal subarachnoid hemorrhage (aSAH) has decreased during the last decades, but most studies have been unable to assess case severities among individual patients. We aimed to assess changes in severity-adjusted aSAH mortality in patients admitted to intensive care units (ICUs).

**Methods:**

We conducted a retrospective, register-based study by using the prospectively collected Finnish Intensive Care Consortium database. Four out of five ICUs providing neurosurgical and neurointensive care in Finland participated in the Finnish Intensive Care Consortium. We extracted data on adult patients admitted to Finnish ICUs with aSAH between 2003 and 2019. The primary outcome was 12-month mortality during three periods: 2003–2008, 2009–2014, and 2015–2019. Using a multivariable logistic regression model—with variables including age, sex, World Federation of Neurological Surgeons grade, preadmission dependency, significant comorbidities, and modified Simplified Acute Physiology Score II—we analyzed whether admission period was independently associated with mortality.

**Results:**

A total of 1,847 patients were included in the study. For the periods 2003–2008 and 2015–2019, the mean number of patients with aSAH admitted per year increased from 81 to 123. At the same time, the patients’ median age increased from 55 to 58 years (*p* = 0.001), and the proportion of patients with World Federation of Neurological Surgeons grades I–III increased from 42 to 58% (*p* < 0.001). The unadjusted 12-month mortality declined from 30% in 2003–2008 to 23% in 2015–2019 (*p* = 0.001), but there was no statistically significant change in severity-adjusted mortality.

**Conclusions:**

Between 2003 and 2019, patients with aSAH admitted to ICUs became older and the proportion of less severe cases increased. Unadjusted mortality decreased but age and case severity adjusted–mortality remained unchanged.

**Supplementary Information:**

The online version contains supplementary material available at 10.1007/s12028-021-01420-z.

## Introduction

Aneurysmal subarachnoid hemorrhage (aSAH) is a devastating form of stroke causing significant mortality and morbidity [1]. The patients benefit from treatment at high-volume centers with specialized neurovascular teams and neurointensive care or similar units [2]. Treatment of aSAH includes several procedures that are best implemented in an intensive care unit (ICU), including reducing the risk of rebleeding through blood pressure control [3, 4], relieving hydrocephalus with an external ventricular drain (EVD), and preventing delayed cerebral ischemia with goal-directed hemodynamic therapy [5].

Randomized controlled trials have shown that, compared with surgical clipping, endovascular coiling of ruptured aneurysms reduces the risk of death and dependency [6]. Accordingly, in the last 10–20 years, endovascular coiling has become the most common treatment modality for ruptured aneurysms [7, 8]. At the same time, studies suggest that case mortality has decreased, both in hospital series [9–12] and population-based [1, 13] studies. The hypothesized factors contributing to this include, in addition to change from clipping to coiling, better diagnostic accuracy of nonsevere aSAH, early aneurysm repair, and improved general medical management. However, the only previous study on solely ICU-treated patients with aSAH contrasted this and did not find any changes in case mortality after 2004, after adjusting for age and illness severity [14].

To clarify the matter, we aimed to assess changes in 12-month mortality after intensive care for aSAH between 2003 and 2019. We hypothesized that severity-adjusted mortality rates had decreased with time.

## Methods

### Study Setting and Population

We conducted a retrospective, register-based study using the Finnish Intensive Care Consortium (FICC) database. The FICC database has previously been described in detail [15]. Briefly, the FICC was established in 1994 as an ICU-benchmarking project, and all data were entered prospectively into the database. Today, all ICUs in mainland Finland, apart from one specialized unit, participate in the FICC. The database is maintained by TietoEVRY (Helsinki, Finland).

Neurosurgery and neurointensive care are provided only at five university hospitals in Finland. Four of these five units providing neurosurgical and neurointensive care (in the university hospitals of Kuopio, Oulu, Tampere, and Turku) participate in the FICC. These hospitals cover approximately two thirds of the population in Finland.

From the FICC database, we extracted data on patients admitted with a diagnosis indicating aSAH between 2003 and 2019 in these four units. aSAH was defined if the patient had an Acute Physiology and Chronic Health Evaluation III diagnosis indicating SAH and an International Classification of Diseases, 10th revision diagnosis of I60.0–I60.7.

We only included adult patients (age ≥ 18 years). We excluded foreigners and nonemergency admissions. Because of the low number of missing data, we excluded patients with missing data.

### Definition of Covariates

We extracted all covariates from the FICC database. Age was measured on admission. The Glasgow Coma Scale (GCS) score was defined as the worst measured GCS score during the first ICU-day or as the last reliable GCS for intubated and/or sedated patients, according to the Simplified Acute Physiology Score (SAPS) II definition [16]. Based on this GCS score, the patients were classified into patients with a good grade (World Federation of Neurological Surgeons [WFNS] grade I–III) and patients with a poor grade aSAH (WFNS grade IV–V) [17].

Preadmission functional status was a modified version of the World Health Organization/Eastern Cooperative Oncology [18] classification used in the FICC. Significant comorbidity was recorded if at least one of the SAPS II [16] or Acute Physiology and Chronic Health Evaluation II [19] comorbidities was present. Placement of an intracranial pressure probe or an EVD, as well as further information on ICU treatment, was obtained through the TISS-76 [20] or TISS-28 [21] recordings. For severity of illness adjustment, we created a modified SAPS II score without age, GCS, comorbidity, and admission type subscores.

Our primary outcome of interest was 12-month mortality. We also report crude hospital mortality rates.

### Statistical Analyses

We used IBM SPSS Statistics for Macintosh (Version 26.0; IBM Corp, Armonk, NY) for the statistical analyses.

We report categorical data as numbers with percentages. We compared categorical data across groups by using a two-sided *χ*^2^ test. None of the continuous variables followed normal distribution according to the Kolmogorov–Smirnov test and visual inspection of histograms. Hence, we report medians with interquartile ranges and compared data across two groups by using the Mann–Whitney *U*-test and across several groups by using the Kruskal–Wallis test. *p* < 0.05 was considered significant in all analyses.

To test the association between admission period and 12-month mortality, we used univariate and multivariable logistic regression, reporting odds ratios (OR) with 95% confidence intervals (CIs). The multivariable model included age, sex, SAH grade (WFNS grades I–III and IV–V), preadmission dependency, significant comorbidity, and the modified SAPS II score. We included age and the modified SAPS II score as continuous variables.

We divided the admission period into three approximately equally long periods: 2003–2008, 2009–2014, and 2015–2019. This division ensured that every period had an adequate number of patients for the analyses. To test whether admission period was independently associated with 12-month mortality, we added the admission period (as a nominal variable) to the multivariable model described above. If this new model explained more of the variance in outcome (i.e., whether the difference between the log-likelihoods between the models was significant), this would suggest that admission period was independently associated with mortality. We report Nagelkerke’s *R*^2^ and Hosmer–Lemeshow (HL) test results, as well as the receiver operating characteristic area under curve (AUC) for both models.

We also calculated standardized mortality ratio (SMR), i.e., observed mortality divided by predicted mortality, for individual years of the follow-up period by using the multivariable logistic regression model without the admission period described above. For data on individual years, we used linear regression to test for a trend, reporting *R*^2^, and *p* values.

The association between admission period and mortality may depend on the treating hospital. Hence, as a sensitivity analysis, we also tested a generalized linear mixed model including the factors of the multivariable model as fixed effects and the treating hospital as a random effect.

We followed the Strengthening the Reporting of Observational studies in Epidemiology statement for reporting of results [22].

### Ethics Approval

The study was approved by the Finnish institute for health and welfare, THL (Dnro THL/1298/5.05.00/2019) and all participating hospitals. The study was approved by the ethics committee of Helsinki University Hospital (194/13/03/02/2014).

## Results

### Study Population and Characteristics

**Fig. 1:**
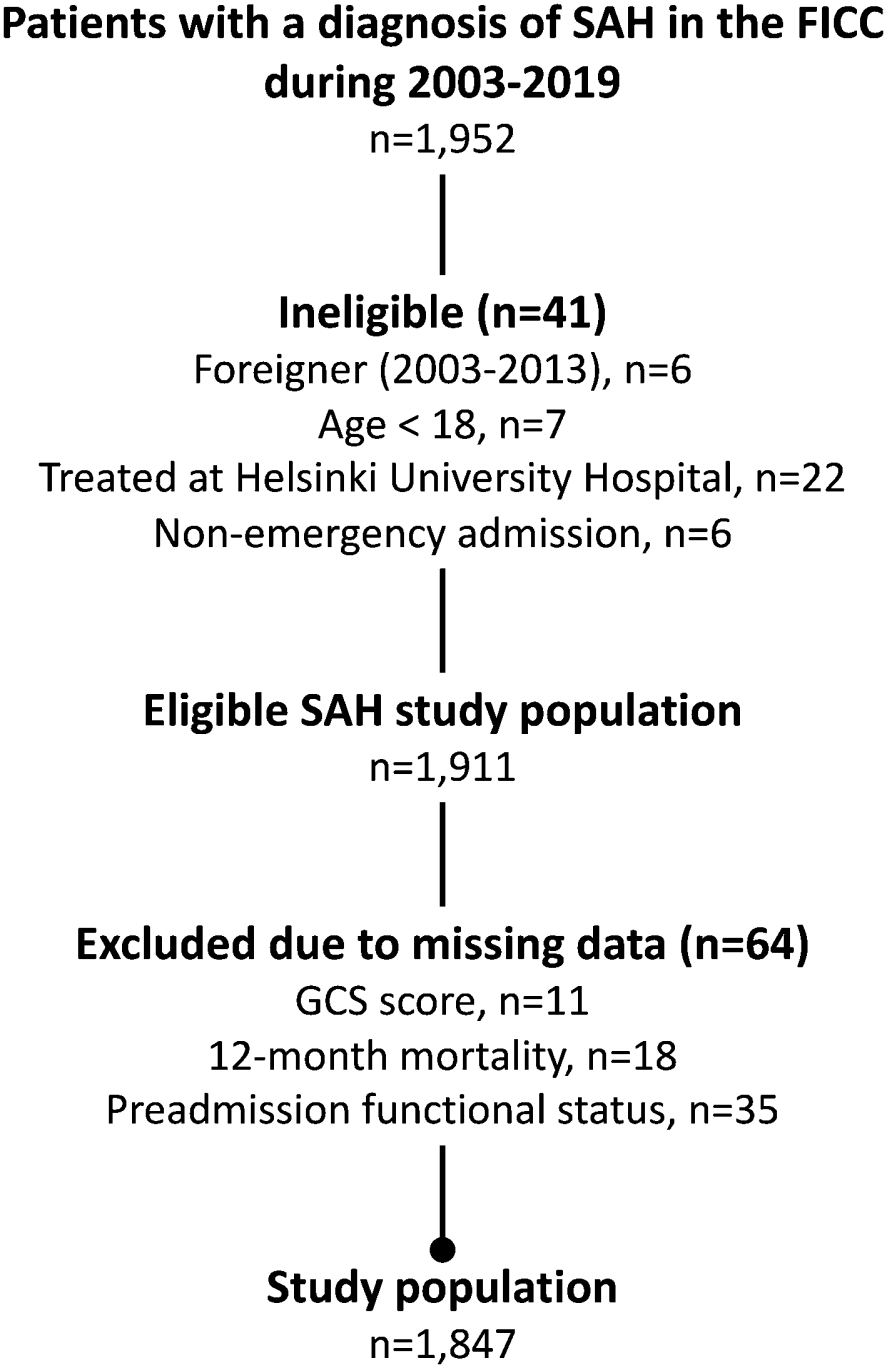
Study flow chart. FICC, Finnish Intensive Care Consortium, GCS, Glasgow Coma Scale, SAH, subarachnoid hemorrhage

A total of 1,952 patients with a diagnosis of aSAH were identified in the FICC. We excluded 41 ineligible patients and 64 patients because of missing data (Fig. 1). Therefore, the final study population included 1,847 patients. The excluded patients had lower GCS scores, higher SAPS II scores, and shorter ICU stays. An intracranial pressure probe or EVD was used more seldom in the excluded patients, and their hospital and 12-month mortality were higher (Supplemental Table 1).

**Table 1:** Characteristics of patients with aneurysmal subarachnoid hemorrhage

Parameters	All patients (*N* = 1,847)	2003–2008 (*n* = 486)	2009–2014 (*n* = 748)	2015–2019 (*n* = 613)	*p* value
Age, median (IQR) (yr)	57 (48–66)	55 (47–64)	56 (48–66)	58 (50–67)	0.001
18–45, *n* (%)	341 (18)	93 (19)	143 (19)	105 (17)	0.56
46–70, *n* (%)	1,210 (66)	324 (67)	486 (65)	400 (65)	
71–95, *n* (%)	296 (16)	69 (14)	119 (16)	108 (18)	
Female sex, *n* (%)	1,067 (58)	265 (55)	429 (57)	373 (61)	0.10
Admissions per year, mean		81	125	123	
Dependent preadmission functional status, *n* (%)	107 (6)	24 (5)	26 (3)	57 (9)	< 0.001
Significant comorbidity, *n* (%)	158 (9)	42 (9)	61 (8)	55 (9)	0.86
GCS, median (IQR)	13 (6–15)	10 (5–14)	13 (6–15)	14 (6–15)	< 0.001
WFNS grade, *n* (%)					< 0.001
I–III	970 (53)	203 (42)	414 (55)	353 (58)	
IV–V	877 (47)	283 (58)	334 (45)	260 (42)	
SAPS II score, median (IQR)	28 (20–47)	32 (23–50)	27 (20–44)	26 (20–47)	< 0.001
ICU treatment, *n* (%)	477 (26)	137 (28)	195 (26)	145 (24)	0.23
ICP monitoring or EVD	477 (26)	137 (28)	195 (26)	145 (24)	0.23
Single vasoactive infusion^a^	609 (33)	178 (37)	235 (31)	196 (32)	0.001
Multiple vasoactive infusions^a^	1,065 (58)	250 (51)	436 (58)	379 (62)	
Central venous catheter	1,309 (71)	422 (87)	537 (72)	350 (57)	< 0.001
Acute dialysis	9 (0.5)	3 (0.6)	4 (0.5)	2 (0.3)	0.77
Enteral nutrition	489 (27)	134 (28)	209 (28)	146 (24)	0.19
Parenteral nutrition	250 (14)	79 (16)	98 (13)	73 (12)	0.10
Tracheostomy^b^		55 (11)	76 (10)		0.52
Length of ICU stay, median (IQR)	2.8 (1.5–6.7)	2.5 (1.3–5.1)	2.9 (1.7–7.0)	2.9 (1.7–6.9)	< 0.001
Mortality, *n* (%)					
Hospital	299 (16)	99 (20)	96 (13)	104 (17)	0.002
12-month	449 (24)	148 (30)	157 (21)	144 (23)	0.001

*p* values for tests between periods are shown

*EVD* external ventricular drain, *GCS* Glasgow Coma Scale, *ICP* intracranial pressure, *ICU* intensive care unit, *IQR* interquartile range, *SAPS II* Simplified Acute Physiology Score II, *WFNS* World Federation of Neurological Surgeons

^a^Includes nimodipine infusion

^b^Only available for 2003–2014

The population characteristics are described in Table 1. Briefly, the median age was 57 years (interquartile range 48–66) and the majority were women (58%). Poor premorbid functional status, i.e., dependence on help in self-care (6%) and significant comorbidities (9%), were rare. Unadjusted hospital and 12-month mortalities were 16% and 24%, respectively.

### Changes in Patient Characteristics During the Study Period

**Fig. 2:**
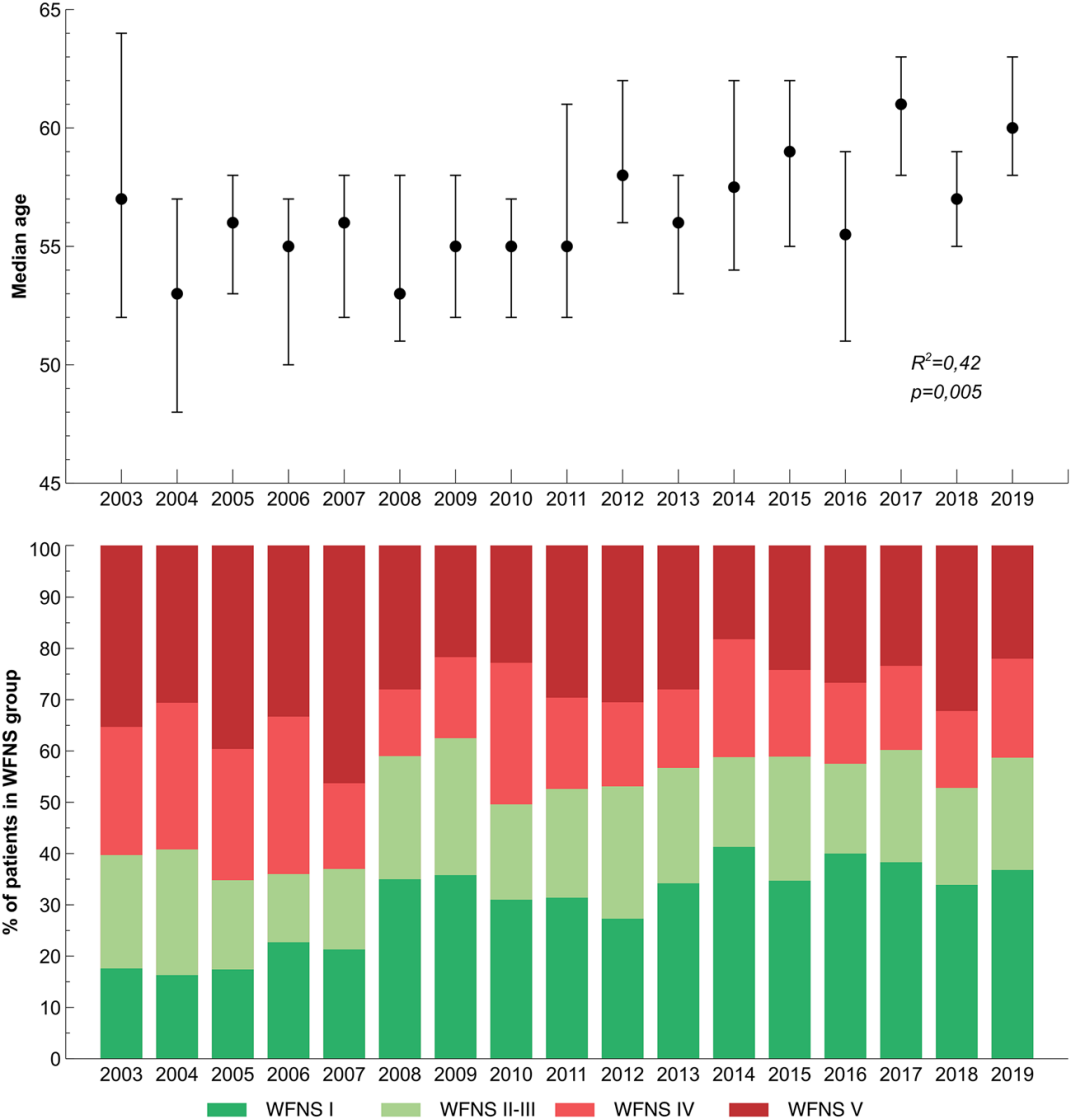
Median ages with interquartile ranges (upper panel), and percentages of patients in WFNS groups (lower panel). *R*^2^ and *p* values are shown for linear regression. WFNS, World Federation of Neurological Surgeons

The mean number of patients admitted per year increased until 2009 and remained stable after that (Table 1, Supplemental Figure). The median age increased between the periods. The proportion of dependent patients increased after 2014. Median GCS scores increased progressively, as well as the proportion of patients with WFNS grade I–III. SAPS II scores decreased and ICU length-of-stays increased (Table 1). Similar results were seen when comparing individual admission years (Fig. 2, Supplemental Figure).

We only had data on the proportion of patients who underwent surgical aneurysm repair in 2003–2013. This proportion decreased from 37% in 2003 to 19% in 2013, without significant steps between any 2 years (*R*^2^ = 0.51 and *p* = 0.013 for linear trend).

### Changes in Mortality and SMR During the Study Period

Hospital mortality decreased from 20% in 2003–2008 to 13% in 2009–2014 and 17% in 2015–2019 (*p* = 0.002). Likewise, crude 12-month mortality was lower in 2009–2014 (21%) and 2015–2019 (23%) than that in 2003–2008 (30%, *p* = 0.001).

In the multivariable model including the admission period, increasing age, WFNS grade IV–V, male sex, and higher modified SAPS score were associated with increased mortality. In contrast, there was no difference between the admission periods in adjusted 12-month mortality (Table 2). The model had a Nagelkerke *R*^2^ of 0.47, HL test *p* = 0.79, and an AUC of 0.88 (95% CI 0.86–0.89), indicating excellent performance.

**Table 2:** 12-month mortality rates and ORs for death at 12 months

Parameters	12-month mortality (%)	Univariate model		Multivariable model	
		OR	95% CI	OR	95% CI
Age (yr)		1.04	1.03–1.05	1.05	1.04–1.06
18–45	11				
46–70	23				
71–95	44				
Sex					
Male	27	Ref		Ref	
Female	23	0.81	0.66–1.01	0.65	0.49–0.86
WFNS grade					
I–III	6	Ref		Ref	
IV–V	45	13.25	9.81–17.91	6.06	4.34–8.45
Preadmission status					
Independent	23	Ref		Ref	
Dependent	40	2.21	1.48–3.30	1.48	0.88–2.49
Significant comorbidity					
No	23	Ref		Ref	
Yes	33.5	1.65	1.16–2.34	1.33	0.84–2.12
Modified SAPS II score		1.20	1.17–1.22	1.15	1.13–1.18
Admission year					
2003–2008	30	Ref		Ref	
2009–2014	21	0.61	0.47–0.79	0.76	0.55–1.05
2015–2019	23	0.70	0.54–0.92	0.86	0.61–1.21

Multivariable model includes age, sex, WFNS grade, preadmission independence status, significant comorbidities, modified SAPS II, and admission year strata. Age and modified SAPS II were included as continuous variables

*CI* confidence interval, *GCS* Glasgow Coma Scale, *OR* odds ratio, *SAPS* Simplified Acute Physiology Score, *WFNS* World Federation of Neurological Surgeons

The multivariable model without admission period had a Nagelkerke *R*^2^ of 0.47, HL test *p* = 0.70, and an AUC of 0.87 (95% CI 0.86–0.89). The two models did not differ in their accuracy (log-likelihoods 1,349 and 1,351, respectively, *p* = 0.25), indicating that admission period was not independently associated with 12-month mortality.

The results did not significantly differ in the generalized linear mixed model including treating hospital as a random effect (Supplemental Table 2). In addition, the results of the multivariable model did not change when including the 35 patients with missing data on preadmission functional status (Supplemental Table 3).

To assess possible changes in mortality in only good-grade (WFNS I–III) patients or poor-grade (WFNS IV-V) patients, we used the multivariable model without WFNS grade separately for both groups. There were no changes in adjusted 12-month mortality in either group, but significant comorbidities were associated with mortality only in good-grade patients. (Supplemental Table 4).

**Fig. 3:**
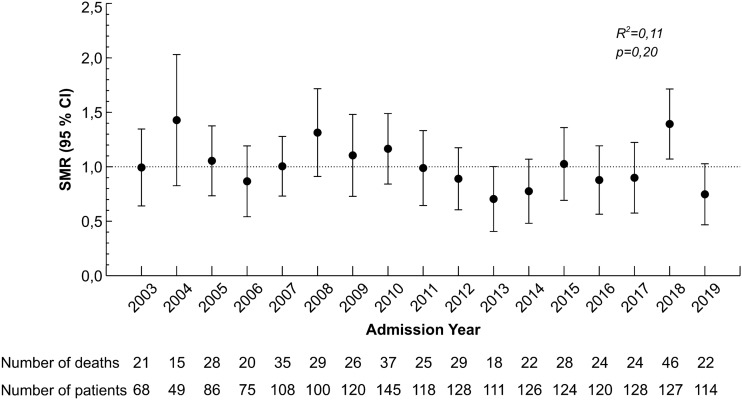
Standardized mortality ratios (SMR) for each year of the study period. *R*^2^ and *p* values are shown for linear regression

SMRs for every year of the study period are shown in Fig. 3. SMR did not change significantly during the follow-up period (*R*^2^ = 0.11, *p* = 0.20).

## Discussion

### Key Findings

From 2003 to 2019, the proportion of ICU-admitted patients with good grade aSAH (WFNS I–III) had increased, while at the same time the admitted patients were older and more often previously functionally dependent. These changes likely reflect two paradigm changes. First, aSAH is recognized as a major indication for specialized neuroscience ICU care, and hence a larger proportion of patients are admitted to an ICU, regardless of clinical severity. This interpretation is further supported by the finding that the number of ICU-treated patients per year increased during our study period, while at the same time the overall incidence of aSAH in Finland has been shown to decrease [23].

Second, the number of active and previously healthy older people has increased, and hence they are more often admitted to ICUs. This is not unique to patients with aSAH, but is seen in all ICU admissions [24, 25]. The adoption of endovascular treatment of ruptured aneurysms has possibly contributed to this as well, and because of its less invasive nature, a greater proportion of patients can be considered for active treatment. Length of ICU stays increased slightly during the study period. Overall, length-of-stays were rather short, but the hospitals had step-down units and specialized wards where patients could be transferred to for further monitoring.

The second major finding of our study is that after adjusting for age, sex, and case severity, no change in 12-month mortality was seen during the study period. In contrast, unadjusted 12-month mortality was lower in 2003–2008 than that in 2009–2019. In addition, the increase in the proportion of good-grade patients with aSAH was the most clear before 2009 and remained somewhat stable after that. In contrast, the patients’ median age and the proportion of patients over 70 years old increased more gradually from 2003 to 2019.

It is worth mentioning that the 12-month mortality of good-grade patients with aSAH was only 6%; therefore, our study was possibly not powered to detect changes in their mortality, and it could be argued that mortality is not an optimal outcome measure for good-grade patients. However, even when taking only poor-grade patients into account, there was no change in the adjusted 12-month mortality during the study period.

### Comparison with Previous Literature

Previous studies based on hospital or population series on patients with aSAH have found decreasing mortality rates, but have reported only unadjusted [1], or age-adjusted and/or sex-adjusted [9–13] results. The only previously published study on ICU-admitted patients with aSAH, by Udy and colleagues [14], reported similar results with our study: despite an absolute reduction of case fatality, after adjustment for age and case severity, year of admission beyond 2003 was not associated with significant change in in-hospital mortality.

Taken together, the overall mortality rate of aSAH has decreased, but because the studies suggesting this have not adjusted for case severity, the studies rather reflect changes in admission criteria and diagnostic accuracy instead of changes in treatment outcomes. In contrast, the severity-adjusted outcomes of ICU-treated patients have not improved, suggesting that the lower overall mortality could be largely due to better diagnostic accuracy of good-grade aSAH and not due to safer aneurysm treatment (i.e., endovascular coiling) or improved medical management.

Spontaneous intracranial hemorrhage (ICH) is another devastating form of hemorrhagic stroke often requiring treatment at an ICU. As for aSAH, most [26–28] but not all [29] recent studies suggest that overall ICH case fatality has decreased over time, but there are no long-term studies that have been able to control for ICH severity.

Previous studies have shown a strong association between the volume of patients with aSAH and outcome. These studies have defined a high-volume center as treating at least 13 to 14 patients with aSAH per year [30, 31]. The lowest volume hospital in our study treated on average 17 patients per year. Thus, differences in hospital volume are not likely to explain the unchanged prognosis. It is worth mentioning that the overall 12-month mortality was 24%, which is the same as that of a national Swiss study [32].

### Strengths and Limitations

Our study has several strengths. We were able to analyze a large, multicenter series of patients treated during a 17-year-long study period. We also had extensive, prospectively collected data on case severity (i.e., GCS and SAPS II), as well as preadmission functional status and comorbidities. In contrast with the previous study by Udy and colleagues [14], we could report 12-month mortality in addition to hospital mortality.

Some limitations of our study should be mentioned. We only included patients with International Classification of Diseases, 10th revision diagnoses I60.0–I60.7, excluding diagnoses I60.8 and I60.9 (i.e., other nontraumatic SAH and unspecified nontraumatic SAH, respectively). The excluded patients likely included some patients who eventually were diagnosed with aSAH. Therefore, one cannot reliably assess the incidence of ICU-treated aSAH based on our data. For the same reason, crude mortality rates may not be representative of all ICU-treated patients with aSAH. Additionally, we only had data on mortality and not on functional outcomes.

Perhaps the largest paradigm change in the treatment of patients with aSAH during our study period has been a move to endovascular treatment for most patients [7, 8]. We had data on the proportion of patients who underwent surgical aneurysm repair in 2003–2013, and this proportion decreased from 37 to 19%. As some aneurysms (e.g., middle cerebral artery bifurcation aneurysms) are still considered to be better suited for surgical treatment, we find it unlikely that the proportion of surgically treated patients has decreased significantly after 2013. We did not have access to individual patients’ imaging findings.

Our results are only valid for patients who survived until hospital admission and were admitted to an ICU and therefore do not reflect all patients with aSAH, as even today a significant proportion of patients die before reaching a hospital [33], and some patients are considered too moribund to justify ICU admission. We included patients only from one European country, and thus we do not know about generalizability compared with other settings. In addition, the excluded patients had lower GCS and higher mortality, which could have been a source of bias, even though inclusion of patients with missing data on preadmission functional status did not affect the results.

Our study population probably includes some patients who were admitted to an ICU solely as potential organ donors, but we were unable to assess the number of such patients. In 2010, Finland adopted an opt-out policy for organ donation, and this might have slightly increased the number of these patients. In 2018–2019, 10% of patients were treated as potential organ donors at some point during their ICU stay (data available only for these years), so we consider it unlikely that the change in legislation significantly affected our results.

## Conclusions

Over time, older and less-severely ill patients with aSAH are admitted to Finnish ICUs, but severity-adjusted treatment results have not changed. Extensive research on aSAH treatment has been done during the last decade [34], but further studies are needed to gain insight into why this appears to not have affected mortality.

## Supplementary Information

Below is the link to the electronic supplementary material.

Supplementary file1 (PDF 56 KB)

Supplementary file2 (PDF 56 KB)

Supplementary file3 (PDF 57 KB)

Supplementary file4 (PDF 59 KB)

Supplementary file5 (PDF 2659 KB)
