# Supplementary material for: Trends in Mortality after Intensive Care of Patients with Aneurysmal Subarachnoid Hemorrhage in Finland in 2003–2019: A Finnish Intensive Care Consortium study
Source: Neurocrit Care. 2021 Dec 29;37(2):447–54. doi: 10.1007/s12028-021-01420-z (PMC9519655; doi:10.1007/s12028-021-01420-z)
Supplement: Supplementary file 1 — Supplementary file1 (PDF 56 KB) [file 12028_2021_1420_MOESM1_ESM.pdf]

Supplemental Table 1

Comparisons between included and excluded patients. P values for tests between groups are shown. None of the continuous variables were normally distributed, so Kruskall-Wallis test was used. Chi-square test was used for categorical variables.

	**Included patients (n=1847)**	**Excluded patients (n=105)**	**p**
**Age**, median (IQR)	57 (48–66)	54 (46–64.5)	0.09
**Female sex**	1067 (57.8 %)	64 (61.0 %)	0.52
**Dependent pre-admission functional status**	107 (5.8 %)	4 (6.2 %)[a]	0.90
**Significant comorbidity**	158 (8.6 %)	3 (2.9 %)	0.04
**GCS**, median (IQR)	13 (6–15)	7.5 (3-15)[b]	0.001
**SAPS II score,** median (IQR)	28 (20-47)	32 (20-57)	0.048
**ICP probe or EVD**	477 (25.8 %)	17 (16.2 %)	0.03
**Length of ICU stays,** median (IQR)	2.8 (1.6-6.7)	2.0 (0.8-4.9)	0.001
**Mortality**			
Hospital	299 (16.2 %)	38 (36.2 %)	<0.001
12-month	449 (24.3 %)	40 (47.1 %)[c]	<0.001

[a] Missing for 40 patients

[b] Missing for 11 patients

[c] Missing for 20 patients

EVD: external ventricular drain, GCS: Glasgow Coma Scale, ICP: intracranial pressure, ICU: intensive care unit, IQR: interquartile range; SAPS II: Simplified Acute Physiology Score II
