# Supplementary material for: Trends in Mortality after Intensive Care of Patients with Aneurysmal Subarachnoid Hemorrhage in Finland in 2003–2019: A Finnish Intensive Care Consortium study
Source: Neurocrit Care. 2021 Dec 29;37(2):447–54. doi: 10.1007/s12028-021-01420-z (PMC9519655; doi:10.1007/s12028-021-01420-z)
Supplement: Supplementary file 2 — Supplementary file2 (PDF 56 KB) [file 12028_2021_1420_MOESM2_ESM.pdf]

Supplemental Table 2

Odds ratios for death at 12-month. The model includes age, sex, WFNS grade, preadmission independence status, significant comorbidities, modified SAPS and admission year, and treating hospital was inserted as a random factor. Age and modified SAPS were included as continuous variables.

	OR	95 % CI
**Age**	1.05	1.04-1.06
**Sex**		
Male	Ref	
Female	0.64	0.54-0.76
**WFNS grade**		
I-III	Ref	
IV-V	6.02	4.35-8.35
**Preadmission status**		
Independent	Ref	
Non-independent	1.49	1.16-1.81
**Comorbidity**		
No	Ref	
Yes	1.32	0.95-1.83
**Modified SAPS II**	1.15	1.14-1.17
**Admission year**		
2003–2008	Ref	
2009–2014	0.77	0.59-1.00
2015-2019	0.90	0.57-1.42

OR: odds ratio, SAPS: Simplified Acute Physiology Score, WFNS: World Federation of Neurological Surgeons
