# Supplementary material for: Trends in Mortality after Intensive Care of Patients with Aneurysmal Subarachnoid Hemorrhage in Finland in 2003–2019: A Finnish Intensive Care Consortium study
Source: Neurocrit Care. 2021 Dec 29;37(2):447–54. doi: 10.1007/s12028-021-01420-z (PMC9519655; doi:10.1007/s12028-021-01420-z)
Supplement: Supplementary file 3 — Supplementary file3 (PDF 57 KB) [file 12028_2021_1420_MOESM3_ESM.pdf]

Supplemental Table 3

Odds ratios for death at 12 months including the 35 patients with missing information on preadmission independence status (total n=1882). The model includes age, sex, WFNS grade, significant comorbidities, modified SAPS and admission year. Age and modified SAPS were included as continuous variables.

	OR	95 % CI
**Age**	1.05	1.04-1.06
**Sex**		
Male	Ref	
Female	0.65	0.50-0.86
**WFNS grade**		
I-III	Ref	
IV-V	6.27	4.50-8.74
**Comorbidity**		
No	Ref	
Yes	1.35	0.85-2.14
**Modified SAPS II**	1.15	1.13-1.18
**Admission year**		
2003–2008	Ref	
2009–2014	0.78	0.56-1.08
2015-2019	0.94	0.66-1.32

OR: odds ratio, SAPS: Simplified Acute Physiology Score, WFNS: World Federation of Neurological Surgeons
