# Supplementary material for: Trends in Mortality after Intensive Care of Patients with Aneurysmal Subarachnoid Hemorrhage in Finland in 2003–2019: A Finnish Intensive Care Consortium study
Source: Neurocrit Care. 2021 Dec 29;37(2):447–54. doi: 10.1007/s12028-021-01420-z (PMC9519655; doi:10.1007/s12028-021-01420-z)
Supplement: Supplementary file 4 — Supplementary file4 (PDF 59 KB) [file 12028_2021_1420_MOESM4_ESM.pdf]

Supplemental Table 4

Odds ratios for death at 12-month in WFNS grade subgroups. The model includes age, sex, preadmission independence status, significant comorbidities, modified SAPS and admission year. Age and modified SAPS were included as continuous variables.

	**WFNS grade I-III (n=970)**		**WFNS grade IV-V (n=877)**	
	**OR**	**95 % CI**	**OR**	**95 % CI**
**Age**	1.07	1.05-1.10	1.04	1.03-1.06
**Sex**				
Male	Ref		Ref	
Female	0.51	0.28-0.93	0.72	0.52-0.98
**Preadmission status**				
Independent	Ref		Ref	
Non-independent	1.26	0.43-3.70	1.60	0.87-2.93
**Comorbidity**				
No	Ref		Ref	
Yes	2.17	1.02-4.63	1.06	0.61-1.84
**Modified SAPS II**	1.11	1.06-1.16	1.16	1.13-1.19
**Admission year**				
2003–2008	Ref		Ref	
2009–2014	0.80	0.42-1.83	0.72	0.50-1.03
2015-2019	0.66	0.30-1.44	0.93	0.63-1.38

OR: odds ratio, SAPS: Simplified Acute Physiology Score, WFNS: World Federation of Neurological Surgeons
