# Supplementary material for: Trends in Mortality after Intensive Care of Patients with Aneurysmal Subarachnoid Hemorrhage in Finland in 2003–2019: A Finnish Intensive Care Consortium study
Source: Neurocrit Care. 2021 Dec 29;37(2):447–54. doi: 10.1007/s12028-021-01420-z (PMC9519655; doi:10.1007/s12028-021-01420-z)
Supplement: Supplementary file 5 — Supplementary file5 (PDF 2659 KB) [file 12028_2021_1420_MOESM5_ESM.pdf]

Supplemental Figure

Characteristics of the subarachnoid hemorrhage patients for each year of the study period. For the number of admitted patients, the total number of patients is shown. For continuous data, medians with interquartile ranges are shown. For categorical data, proportions with 95 % confidence intervals are shown. R^2 values, p values and beta-coefficients (with 95 % confidence intervals) are shown for linear regression.

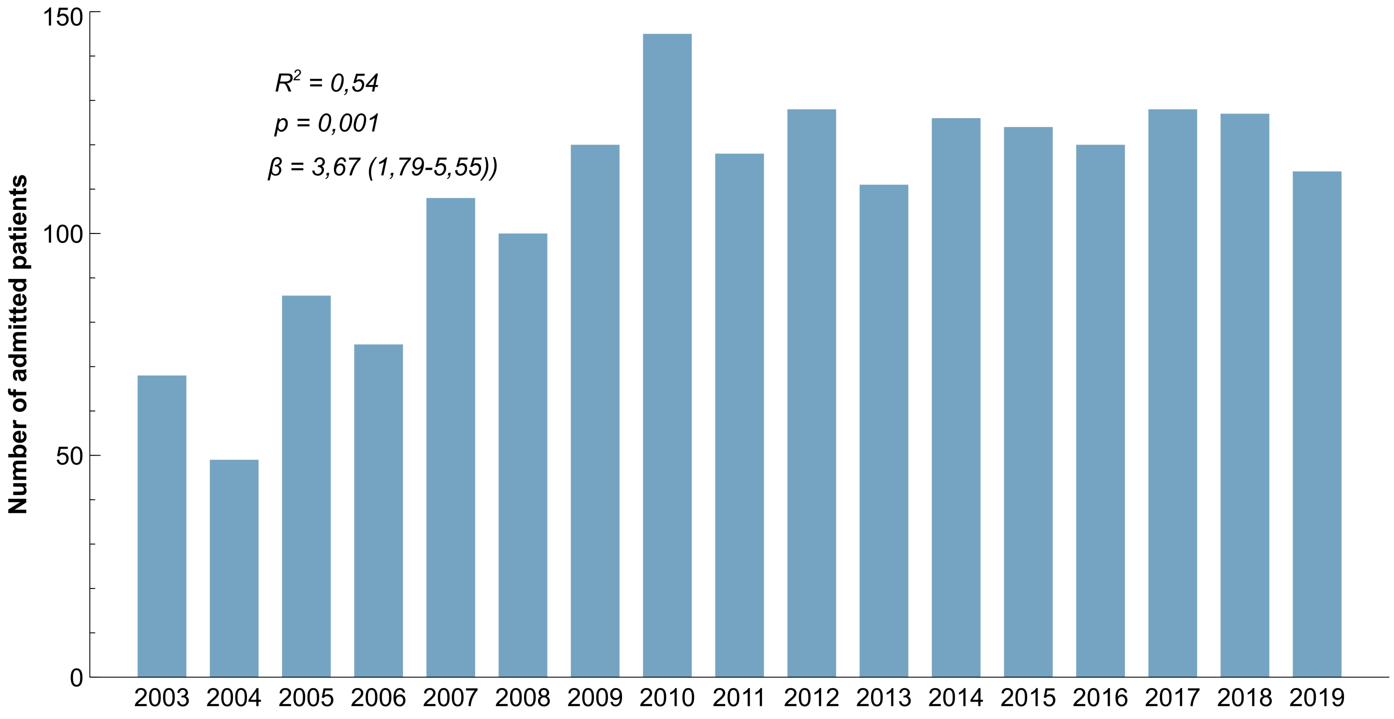

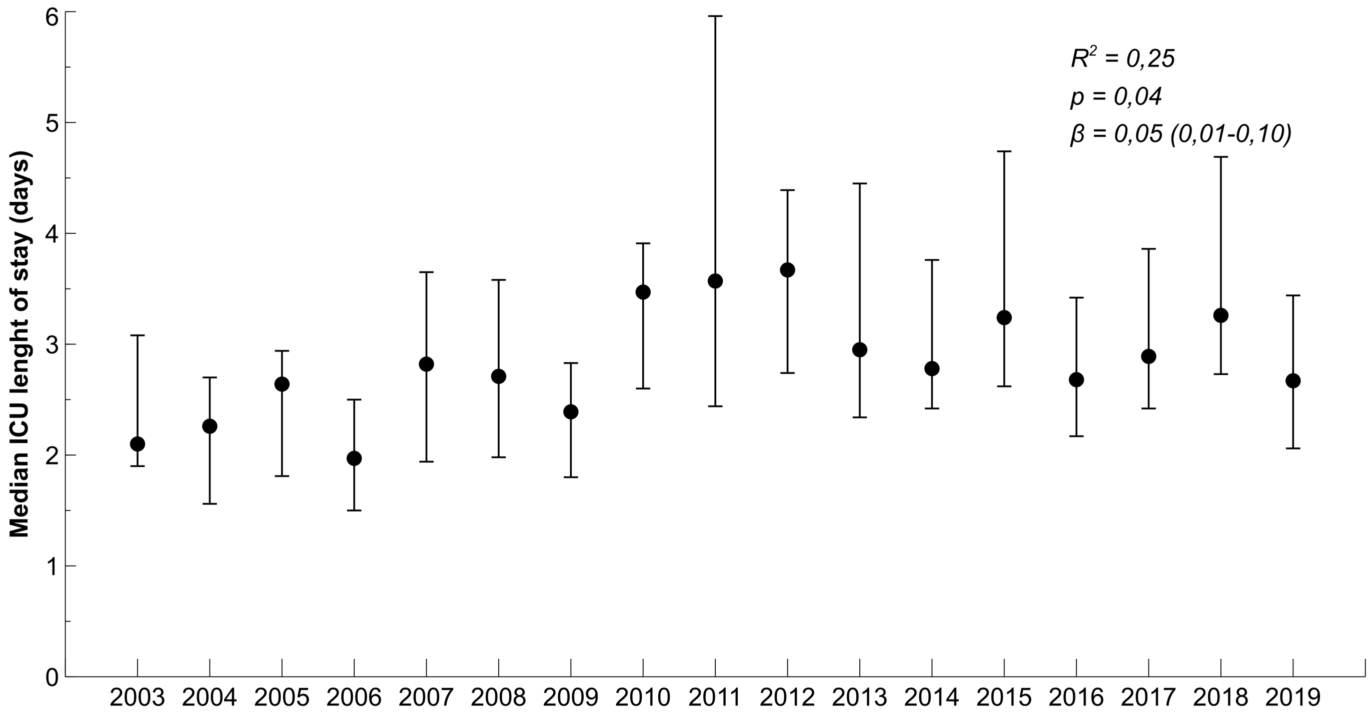

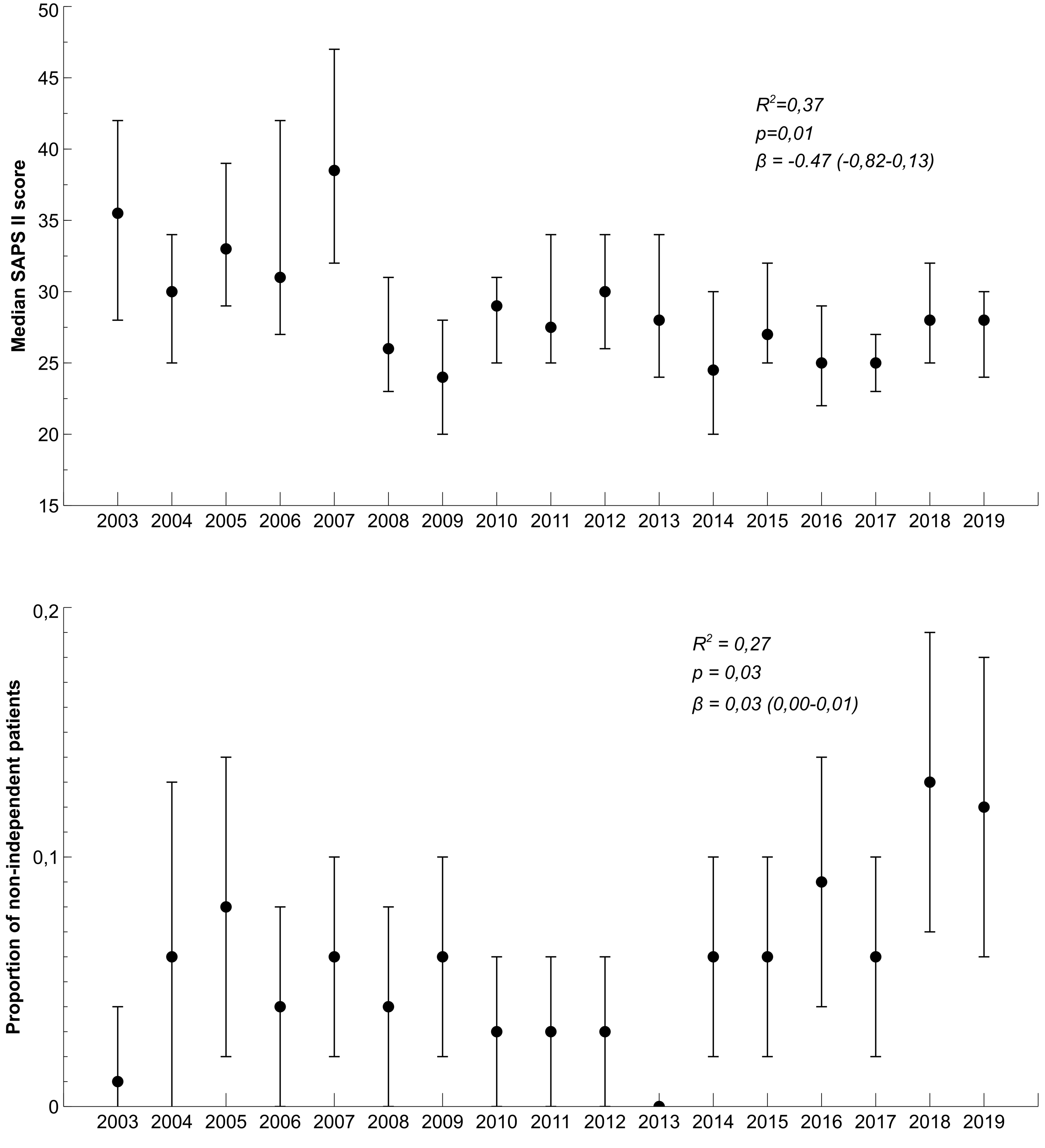
Median SAPS II score
R^2=0,37
p=0,01
β = -0.47 (-0,82-0,13)
50
45
40
35
30
25
20
15
2003 2004 2005 2006 2007 2008 2009 2010 2011 2012 2013 2014 2015 2016 2017 2018 2019
Proportion of non-independent patients
R^2 = 0,27
p = 0,03
β = 0,03 (0,00-0,01)
0,2
0,1
0
2003 2004 2005 2006 2007 2008 2009 2010 2011 2012 2013 2014 2015 2016 2017 2018 2019
